# Development of WO_3_–Nafion Based Membranes for Enabling Higher Water Retention at Low Humidity and Enhancing PEMFC Performance at Intermediate Temperature Operation

**DOI:** 10.3390/polym14122492

**Published:** 2022-06-19

**Authors:** Asmaa Selim, Gábor Pál Szijjártó, Loránd Románszki, András Tompos

**Affiliations:** 1 Research Centre for Natural Sciences, Renewable Energy Group, Institute of Materials and Environmental Chemistry, Magyar Tudósok Körútja 2, H-1117 Budapest, Hungary; szijjarto.gabor@ttk.hu (G.P.S.); tompos.andras@ttk.hu (A.T.); 2 National Research Centre, Chemical Engineering and Pilot Plat Department, Engineering and Renewable Energy Research Institute, 33 El Bohouth Street, Giza 12622, Egypt; 3 Research Centre for Natural Sciences, Functional Interfaces Research Group, Institute of Materials and Environmental Chemistry, Magyar Tudósok Körútja 2, H-1117 Budapest, Hungary; romanszki.lorand@ttk.hu

**Keywords:** proton exchange membranes, hybrid inorganic–organic membranes, Nafion, low humidity fuel cells, tungsten oxide, hydration degree, mechanical stability

## Abstract

The proton exchange membrane (PEM) represents a pivotal material and a key challenge in developing fuel cell science and hydrogen technology. Nafion is the most promising polymer which will lead to its commercialisation. Hybrid membranes of nanosized tungsten oxide (WO_3_) and Nafion were fabricated, characterised, and tested in a single cell. The incorporation of 10 wt% WO_3_ resulted in 21% higher water uptake, 11.7% lower swelling ratio, almost doubling the hydration degree, and 13% higher mechanical stability of the hybrid membrane compared to the Nafion XL. Compared to commercial Nafion XL, the rNF–WO-10 hybrid membrane showed an 8.8% and 20% increase in current density of the cell at 0.4 V operating at 80 and 95 °C with 1.89 and 2.29 A/cm^2^, respectively. The maximum power density has increased by 9% (0.76 W/cm^2^) and 19.9% (0.922 W/cm^2^) when operating at the same temperatures compared to the commercial Nafion XL membrane. Generally, considering the particular structure of Nafion XL, our Nafion-based membrane with 10 wt% WO_3_ (rNF–WO-10) is a suitable PEM with a comparable performance at different operating conditions.

## 1. Introduction

Since ancient times, humans have been using the closest and most easily available energy sources in massive quantities. Those sources are nonrenewable and nonenvironmentally friendly and are usually associated with several environmental–civilisational problems. Nowadays, our generation’s challenge is finding and developing new clean, renewable, and environmental-friendly energy sources, which could help stop the consumption of the existing sources while reducing the prevalent environmental problems, such as sustainable energy [1,2,3].

Hydrogen energy technologies and fuel cells may play a substantial role as sustainable energy techniques relying on renewable energy. Hydrogen is the main raw material for the PEMFC, and it is possible to obtain hydrogen by splitting water using renewable energy, even if this process is inherently intermittent. The vast majority of renewable energy sources are intermittent, which creates gaps in both space and time between the availability of the energy and the end-users’ ability to consume it. It is important to develop acceptable energy storage and generating systems for the electrical grid in order to handle these difficulties [4].

Using a fuel cell enables the conversion of the chemical energy stored in the fuel into electrical energy directly with only water and heat as by-products. That makes fuel cells act as a favourable source of renewable clean energy [5,6,7].

Based on the material and fuel type, several fuel cells have been reported, such as proton exchange membrane (polymer electrolyte) fuel cells (PEMFCs) [8,9,10,11], direct methanol fuel cell (DMFCs) [12,13,14], alkaline fuel cells (AFCs) [15,16,17], and solid oxide fuel cells (SOFCs) [18,19]. Among the fuel cells, PEMFC technologies have received worldwide intense attention due to their high efficiencies, low emissions, and high power density. PEMFCs are also in the spotlight due to their fast start-up because of the low temperature, which gives them the advantage of being portable and mobile power generation apparatus. Membrane electrode assemblies (MEAs) have two key components: a) PEM (polymer electrolyte membrane), which acts as an electrolyte and is responsible for transferring the protons from the anode to the cathode and preventing the electrons, and b) the electrocatalyst for the reaction, usually a Pt-based material [3,6,20,21].

To date, several polymers have been used for PEM fuel cell membrane fabrication: perfluorinated polymers such as Nafion [22], nonfluorinated ones such as sulfonated poly(ether ether ketone) [14,23,24], and poly(benzimidazole) [25,26], and even other types of polymers, such as poly (vinyl alcohol) [27,28,29], polyvinylidene difluoride [30,31], and modified cellulose [32]. Nafion is popular due to its acceptable chemical and mechanical stability, and, most importantly, high proton conductivity at low and intermediate temperatures.

The high conductivity of Nafion is attributed to the presence of SO_3_H groups which help the proton conduction across the hydrated membrane. In addition, the existence of the hydrophobic backbone based on perfluorinated polytetrafluoroethylene (PTFE) facilitates the proton transport. Eventually, the PTFE backbone is not only responsible for the morphological and mechanical stability, but also plays a crucial role in providing Nafion with broad channels due to the phase separation from the hydrophilic phase [33]. Nevertheless, not only protons but also hydrogen molecules permeate easily through the clusters containing water.

Hydrogen permeability through hydrated Nafion is known to be significantly higher as compared to its dry state. This is because of the plasticisation effect of water in the Nafion chain decreasing the glass transition temperature of the Nafion polymer. This phenomenon, known as hydrogen crossover, leads to the build-up of mixed potentials, which negatively affects the fuel cell efficiency by lowering the current density and facilitating the formation of H_2_O_2_ [34,35]. This emphasises the importance of membrane hydration, which should be maintained to an optimum level so not to lose the proton conductivity of the polymeric membrane. Although inserting humidified gases externally could help overcome this problem, it results in increasing the cell size, which is a barrier to commercialisation [36,37,38,39].

To overcome the above issues, modifying the microstructure of the hydrophilic sulfonic group and the hydrophobic fluoric groups in the Nafion backbone by inserting different organic or inorganic fillers/components, and hence fabricating the so-called composite or hybrid membrane, became one of the most promising approaches [40,41,42]. Significant effort has been made to fabricate composite membranes based on Nafion polymers in order to control the membrane hydration at a low relative humidity. Namely, PVA and PTFE have been widely used as polymers to be blended with Nafion to enhance its strength and mechanical stability. On the other hand, a wide variety of inorganic fillers have been used, especially fillers with hygroscopic properties, including metal oxides such as silicon oxide [43,44], titanium oxide [22,45], and zirconium oxide [46,47], nanostructured clay such as laponite [48] and montmorillonite [49], and carbon-based material such as graphene oxide [50,51] and carbon nanotubes [13]. Additionally, cerium oxide has been used to interact with the generated hydrogen peroxide and other reactive hydroxyl and superoxide radicals, which were generated at low temperatures to improve the Nafion’s durability against these radicals and prevent Nafion degradation, and thus the power loss of the fuel cells [52]. Among the metal oxides, Tungsten oxide (WO_3_) is stable in sulphuric acid media, and its effect on the performance of Nafion has been scarcely investigated.

Additionally, WO_3_ can provide (a) a higher hydration level in the membrane during the fuel cell process and (b) greater ionic conductivity for the Nafion when operating at lower humidity or higher temperatures. The number of studies reporting the incorporation of the WO_3_ nanofiller in the Nafion matrix for the PEM fuel cell application is very limited [53,54].

**Figure 1:**
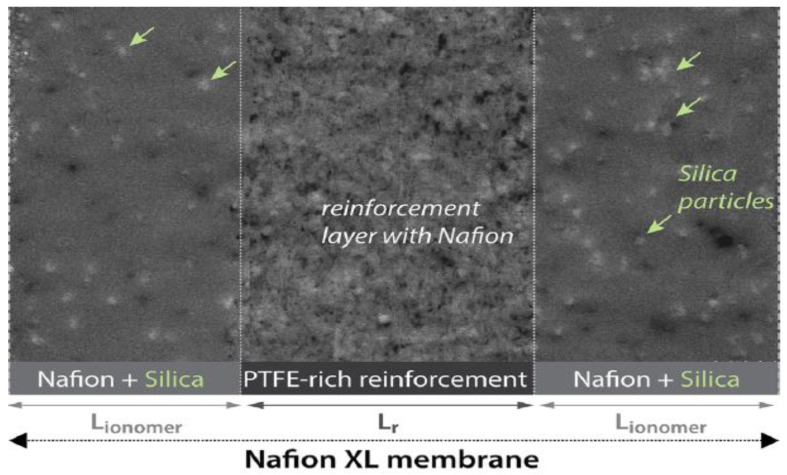
STEM image of the baseline Nafion XL membrane showing the reinforcement layer and additives (Reprinted/adapted with permission from [55] 2022, Asmaa Selim).

In this work, we aimed to study the effect of incorporating hydrothermally synthesised WO_3_ in different concentrations into the Nafion membrane on water uptake, swelling degree, contact angle, thermal stability, conductivity, degree of hydration, and ion exchange capacity. Additionally, the performance of the single H_2_/O_2_ cell was studied at temperature ranges from 25 to 95 °C and the fixed low relative humidity of 50% and 30% for H_2_ and O_2_, respectively. The performance of the produced hybrid membranes is compared with the commercial Nafion XL membrane, which contains SiO2 nanoparticles and has a structure as shown in Figure 1.

## 2. Experimental Methodology

### 2.1. Materials

DuPont Nafion solution (D520-1000 EW) containing 5 wt% copolymer resin was purchased from the fuel cell store. A commercial Nafion XL membrane with a thickness of ca. 27.5 μm was purchased from Ion Power GmbH. Sodium tungstate (Na_2_WO_4_·2H_2_O) and sodium chloride (NaCl) were obtained from CARLO ERBA reagent, EmmendingenGermany. The 2-propanol 99.99% was purchased from Molar Chemicals Kft, Budapest, Hungary. Hydrochloric acid (HCl) 37% and dimethyl acetamide (DMAc) were obtained from VWR Chemicals, Budapest, Hungary. Carbon paper type H23C6 for the Gas Diffusion Electrode (GDE) preparation was obtained from Freudenberg FCCTSE&CO, Germany. Catalyst Powder C-40-PT containing 40% Pt loading was purchased from QuinTech, Göppingen, Germany. Deionized (DI) water (Millipore), used for all membrane preparation, was obtained in-house.

### 2.2. Synthesis of WO_3_ Nanoparticles

The tungsten oxide was synthesised by a hydrothermal method. Briefly, an equal mass ratio of Na_2_WO_4_·2H_2_O (as tungsten precursor) and NaCl (as directing agent) were dissolved in 50 mL of DI water using magnetic stirring. Then, the pH of the solution was adjusted to 2 by the dropwise addition of 6 M HCl. The solution was stirred vigorously for approximately 10 min and then was transferred to a 60 mL Teflon-lined stainless-steel autoclave. Subsequently, the autoclave was sealed and maintained at 180 °C for 24 h, followed by natural cooling to room temperature. After that, the product was washed 3 times with DI water, dried at 60 °C for 12 h, and finally annealed in air at 700 °C for 3 h.

### 2.3. Preparation of Nafion Nanocomposite Membranes

Solution casting method was used for all hybrid membranes. A certain amount of 5 wt% Nafion solution was totally dried at 80 °C in order to evaporate the low-boiling-point solvents and to form a resin. A suitable amount of the resin was then dissolved in DMAc and mixed under a magnetic stirrer until a clear, transparent solution was formed. To this solution, different amounts of the synthesised tungsten oxide were added. The suspension was then treated in an ultrasonic bath for 2 h followed by mixing under vigorous stirring overnight to obtain a uniform dispersion. The casting solution was obtained from 5% Nafion–DMAc with different contents of WO_3_ (0, 5, 10, and 15 wt% WO_3_ with respect to the dry polymer weight). Then, solutions were cast onto glass Petri dishes, and the solvent was evaporated at 80 °C for 24 h. Consequently, the membranes were annealed for 4 h in an oven at 120 °C. The recast Nafion solution membrane and the hybrid membranes were finally obtained by immersing in DI water for a couple of minutes at room temperature. The final membranes were named rNF for the recast Nafion and rNF–WO-x for the hybrid membranes, where x is the weight percent of the added tungsten oxide.

### 2.4. Membrane Characterisation

Morphology and surface characterisation of tungsten oxide nanopowder, recast Nafion, and tungsten hybrid Nafion-based membranes were carried out using a Zeiss EVO 40 XVP scanning electron microscope (SEM) with accelerating voltage of 20 kV, W filament, and a working distance of ~8 mm.

The X-ray diffraction of the synthesised tungsten oxide powder, as well as recast Nafion and hybrid membranes, were recorded using a Philips model PW 3710-based PW 1050 Bragg–Brentano parafocusing goniometer using Cu Kα radiation (λ = 0.15418 nm), with 2*θ* in the range of 4°–75°. Lattice parameters were determined using a full profile fit (Pawley-fit).

The thermal stability of the pure Nafion membrane and the rNF–WO-x membranes were investigated using a TGA-DTA apparatus (model Q500-TA Instrument, Champaign, IL, USA). The temperature was changed between 23 °C–600 °C under a nitrogen atmosphere and a heating rate of 10 °C/min.

A universal testing apparatus (Zwick Z005 GmbH & Co. KG, Ulm, Germany) was used to obtain the mechanical properties of all membranes with a dimension of 75 mm × 10 mm and speed of 20 mm/min, and an initial grip distance of 35 mm.

Fourier transform infrared spectroscopy (FTIR, Tensor II instrument, Bruker, Germany) over the range of 400–4000 cm^−1^ in the attenuated total reflectance (ATR) mode was used to examine the interaction between the tungsten oxide and Nafion polymer chains. FTIR spectra analyses of each membrane were acquired with an average of sixteen scans.

To measure the water uptake and swelling ratio of the membranes, square samples of the membranes with an area of 2.25 cm^2^ were immersed in DI water for 24 h. Then, the surface water was removed with tissue paper. Subsequently, the wet mass and the wet dimensions of the membranes were measured. After that, the samples were dried in a vacuum oven at 50 °C overnight, and the dry weight and size of all samples were measured. Water uptake and swelling ratio were calculated from Equations (1) and (2), respectively [56]:

(1) $$WU=\frac{W_{w}-W_{d}}{W_{d}}\times 100\%$$

where *W_w_* and *W_d_* are the weights of wet and dry membrane samples, respectively.

(2) $$SR=\frac{D_{w}-D_{d}}{D_{d}}\times 100\%$$

where *D*_w_ and *D*_d_ are the size of the wet and dry membrane samples, respectively;

(3) $$D=\sqrt[{2}]{\left(l\times w\right)}$$

where l and w are the length and width of membranes (cm), respectively.

For contact angle (CA) measurements, the samples were mounted on microscope slides with double-sided adhesive tape stripes and placed on the stage of a home-built contact angle goniometer [57]. Ultrapure water (MilliQ, *ρ* = 18.2 MΩ cm) was used as a measuring liquid. To place the water droplets, a 25 µL microsyringe equipped with a PTFE-coated 26 s gauge 3T point style (perpendicular cut) removable needle RN (Hamilton) was used. The microsyringe was operated by a syringe pump (Legato 111, KD Scientific, Holliston, MA, USA). For measuring the advancing CAs, the water was disposed in 1 µL steps up to a volume of 8 µL. To measure receding CAs, the droplet volume was decreased from 8 µL in 1 µL steps. Still images were captured after each 1 µL step. The CAs were determined using the spherical cap approximation for the droplet shape, from the ratio of the droplet image height *h* and base width *w*, according to the formula:

(4) $$\theta =2arctan\left(\frac{2h}{w}\right)\;$$

Each sample was measured at three different locations on its surface. The mean of the same volume droplet CAs were plotted with standard error (SE) for each sample.

To determine ion exchange capacity (IEC) and degree of hydration degree (λ), membranes were fully dried in an oven at 80 °C overnight, and their weight was recorded. Subsequently, the samples were cut into small pieces and soaked in a small beaker with 50 mL 1 M NaCl solutions for 24 h under continuous magnetic agitation. Then, the sample solutions were titrated with 0.01 M NaOH solution using methyl orange as an indicator. The IECs were calculated by the following Equation [58]:

(5) $$IEC=\frac{C_{NaOH}\times V_{NaOH}}{M_{d}}\times 100$$

where $c_{NaOH}$ = 0.01 M, $V_{NaOH}$ is the volume of the NaOH solution used for titration, and $M_{d}$ is the initial dry weight of the membrane.

The hydration degree (λ), the number of water molecules available per SO_3_H group, was determined from Equation (6) [1].

(6) $$\lambda =\frac{10\times WU}{IEC\times M_{wt}}$$

where $M_{wt}$ is the water molecular weight.

Membrane proton conductivity was obtained by applying potentiostatic electrochemical impedance spectroscopy (PEIS) with a frequency from 100 kHz to 10 mHz and a 10 mV amplitude of the oscillating voltage at room temperature. Gas flow was 200 mL N_2_ on the cathode side and 200 mL H_2_ on the anode side. In situ impedance spectra were recorded by connecting the fuel cell with VMP-300 multichannel potentiostat (BioLogic) by contacting the fuel cell cathode to the working electrode and the anode to the reference and the counter electrodes [54]. EC-lab program of BioLogic was applied to carry out and evaluate the PEIS measurements. The membrane resistance was calculated from the low intersect of the Nyquist plot with the *z*-axis. Subsequently, the conductivity of the membranes (S cm^−1^) can be obtained from the following Equation:

(7) $$\sigma =\frac{L}{R\times A}$$

where *L* and A are the thickness and area of the membrane in cm and cm^2^, respectively, while *R* is the membrane resistance in Ω.

### 2.5. MEA Fabrication and Fuel Cell Tests

#### 2.5.1. Catalyst Preparation

QuinTech C-40-PT with 40 m/m% Pt content was used as a reference catalyst, mixed with Nafion solution (5 m/m%) and 2-propanol. The obtained catalyst ink was painted onto the surface of the GDE (4 cm^2^) by a spray coating method using AB200 type airbrush (Conrad Electronic SE). In this work, cathode and anode had the same Pt content (0.15 mg/cm^2^). Afterwards, the GDE was heat-treated in air for 30 min at 80 °C, followed by additional 30 min at 120 °C.

#### 2.5.2. Fuel Cell Tests

In order to obtain the final MEA, the membranes were cut into 16 cm^2^ samples and hot-pressed between the cathode and anode side GDE under 59.4 kg cm^−2^ pressure for 3 min at 120 °C. The resulting MEAs were activated under 400 mV at 80 °C for 4 h before the tests. First, electrochemical impedance spectroscopy measurements were done (for the PEIS conditions, see Section 2.4), and CVs were recorded using the anode side as the reference electrode

The *U*–*I* polarisation plots were recorded at an operating temperature of 25, 60, 80, and 95 °C, under a relative humidity of 50% and 30%, and back pressures of 250 kPa and 230 kPa at the anode and cathode, respectively. The flow rate was equally 200 mL/min for both gases. The polarisation measurements were based on the voltage pulse method reported in our previous work [59]. For comparison, the Nafion XL membrane was tested under the same operating conditions.

## 3. Results and Discussion

### 3.1. Morphology and Properties

**Figure 2:**
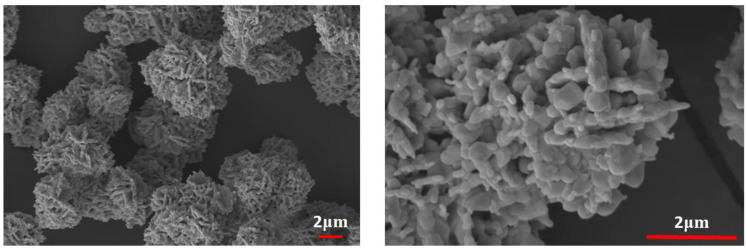
SEM images of the hydrothermally synthesised WO_3_ nanofiller.

**Figure 3:**
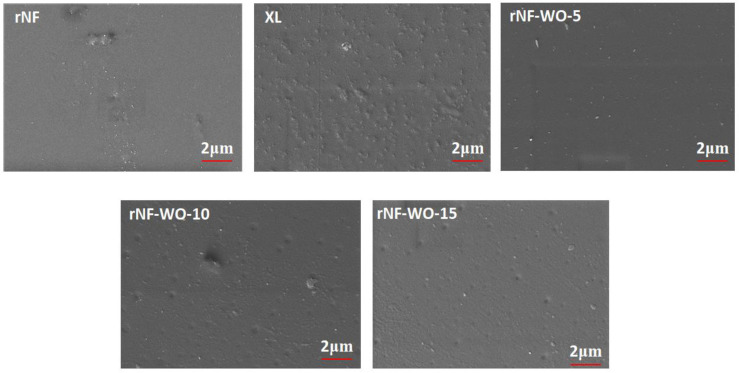
Surface morphology of the hybrid membranes with 5, 10, and 15% WO_3_.

The scanning electron micrograph for the synthesised tungsten oxide nanopowder is shown in Figure 2. WO_3_ nanoparticles were found to vary in size and shape and were slightly aggregated. Nevertheless, the surface of the tungsten-oxide-filled recast Nafion (rNF) membrane was smooth and dense, while the Nafion XL membrane exposed silica nanoparticles (Figure 3).

Similarly, the hybrid membranes containing different WO_3_ concentrations in the casting solution showed dense, compact structures with no voids or cracks. Increasing the concentration from 5 to 10% resulted in protrusions and lumps on the surface of the membranes, only partially present at 15%. However, the surface of the hybrid Nafion–WO_3_ membranes with different WO_3_ concentrations was denser and rougher compared to the recast Nafion membrane (rNF).

**Figure 4:**
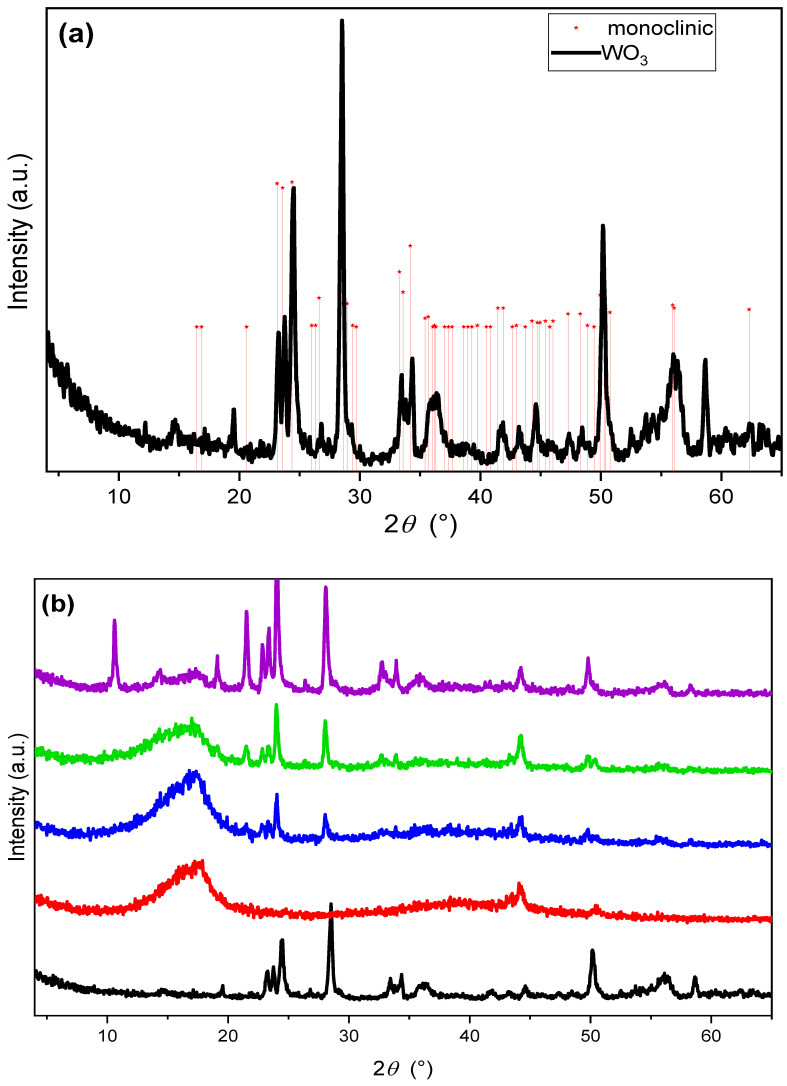
XRD patterns of the synthesised WO_3_ (**a**) and the recast Nafion and hybrid membranes (**b**). Tungsten oxide (black), recast Nafion membrane (red), and Nafion membranes with 5, 10, and 15% tungsten oxide (blue, green, and purple, respectively). Marked with stars are the diffraction peaks from monoclinic WO_3_ (JCPDS no.72-067 7).

The XRD patterns in Figure 4a,b show the degree of crystallinity of the tungsten oxide powder, recast Nafion membrane, and hybrid Nafion–WO_3_ membranes. The formation of tungsten oxide was confirmed, taking into account the monoclinic WO_3_ with lattice parameters *a* = 7.306 Å, *b* = 7.54 Å, and *c* = 7.692 Å align space group P21/c (14) (JCPDS no.72-0677). The obtained WO_3_ pattern correspond to the reported values of the monoclinic WO_3_, with the peaks from other crystallographic phases being absent. For the recast Nafion (rNF) membrane, diffraction peaks around 2θ=17.6° and 44.2° could be observed, representing the typical crystalline phase of Nafion [47,60]. The successful incorporation of the nanofiller in the membranes was evidenced by the detection of the characteristic WO_3_ peaks. Additionally, the sharpening of the peak at 2θ=17.6° after the incorporation of 5 and 10% WO_3_ in the membrane indicates an enhancement of the crystallinity of Nafion. As reported by Shao et al., [53], an increased crystallinity results in improvements in the mechanical stability.

However, the membrane diffraction peaks totally changed after incorporating a higher amount of WO_3_ (15 wt%). A new peak at around 2θ=10.6° can be observed, which could be attributed to the hydrated phase of WO3, possibly formed due to the water-containing channel of the Nafion membrane [61]. The pattern of the rNF–WO-15 shows all the characteristic peaks for the synthesised WO3, suggesting that the membrane accommodates larger size WO_3_ crystallites well-dispersed in the Nafion matrix. Additionally, the peak at 2θ=17.6° is slightly diminished, indicating that the polyfluorocarbon chains in the Nafion membranes, which overlapped the X-ray scattering from the amorphous region of the membrane at lower Bragg angles, have been corrupted upon the addition of a higher amount of WO3, which would probably affect the membrane’s general performance [62].

**Figure 5:**
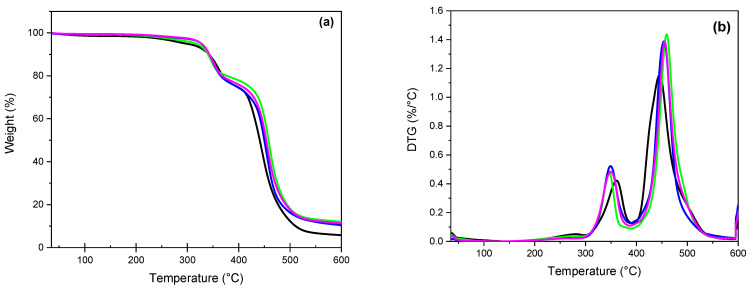
Thermal gravimetric behaviour of recast Nafion and hybrid membranes. Recast Nafion membrane (black) and Nafion membrane with 5, 10, and 15 wt% tungsten oxide (green, blue, and purple, respectively), (**a**) TGA and DTG (**b**).

The thermal stability of the recast Nafion and hybrid Nafion–WO_3_ membranes with different concentrations was investigated and studied using thermogravimetric analysis under N_2_ atmosphere (Figure 5). First, all membranes showed a small weight loss between ca. 50 and 250 °C, which is related to the removal of water (Figure 5b). A more pronounced weight loss indicating the degradation of Nafion can be seen between 300 and 400 °C. The degradation temperatures shifted to a lower temperature for the hybrid membranes compared to the pure Nafion membrane. The unfilled recast membrane started to decompose at around ~320 °C, whereas the hybrid membranes decomposed at ~290 °C; the decomposition peak maxima being around 360 and 330 °C, respectively. This indicates that the incorporation of WO_3_ in the Nafion accelerates the decomposition of the membrane, probably through the deterioration of thermal stability of the SO_3_H groups. As reported, this mass loss is attributed to the decomposition of the acid SO_3_H groups of Nafion, which starts at ~280–300 °C and lasts up to 370–400 °C [53,63]. The further weight loss observed above 400 °C corresponds to the decomposition of the polytetrafluoroethylene backbone chains in the Nafion polymer. In this case, the decomposition temperature of the hybrid membrane appears to be higher than that of the recast membrane, which indicates a stabilising effect of the WO_3_ nanofiller. For all the hybrid membranes, the weight residue is higher compared to the recast Nafion membrane.

**Figure 6:**
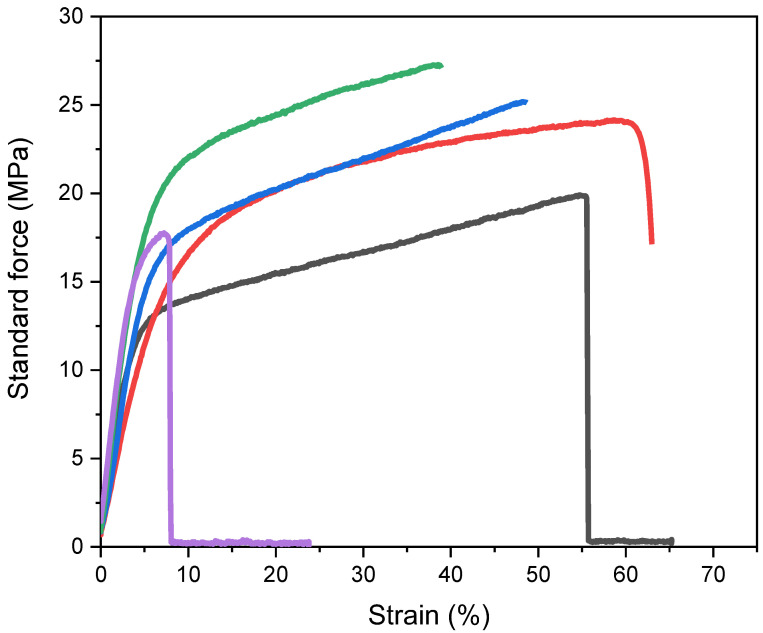
Tensile properties of the membranes; Nafion XL commercial membrane (red), recast Nafion membrane (black), and Nafion membranes with 5, 10, and 15 wt% tungsten oxide (blue, green, and purple, respectively).

Mechanical strength is one of the important properties of a membrane. As reported, the formation of hybrid membranes containing nanosized inorganic fillers is a powerful and easy way to enhance polymer strength [64]. To investigate the effect of tungsten oxide as a nanofiller on the mechanical properties of the Nafion membrane, a tensile test was accomplished. The results are summarised in Figure 6. The recast Nafion had the lowest stress force of a maximum stress of 19.9 MPa. In comparison, the presence of the WO_3_ nanofiller up to 10 wt% resulted in an increase in the membrane strength (25.1 and 27.3 MPa for rNF–WO-5 and rNF–WO-10, respectively). However, incorporating more WO_3_ (15 wt%) led to a dramatic decrease in both the membrane strength and elongation at break at a maximum stress of 17.7 MPa. This might be attributed to the too large crystallite size of the nanoparticles between the Nafion polymer chains [65], as evidenced by XRD. The tensile strength of the rNF–WO-5 and rNF–WO-10 hybrid membranes was higher than that of the Nafion XL membrane. However, the Nafion XL showed a higher elongation at break compared to all prepared membranes. This can be attributed to the structure and composition of the Nafion XL, such as the reinforcement layer and the silica nanoparticles as additives [55].

**Figure 7:**
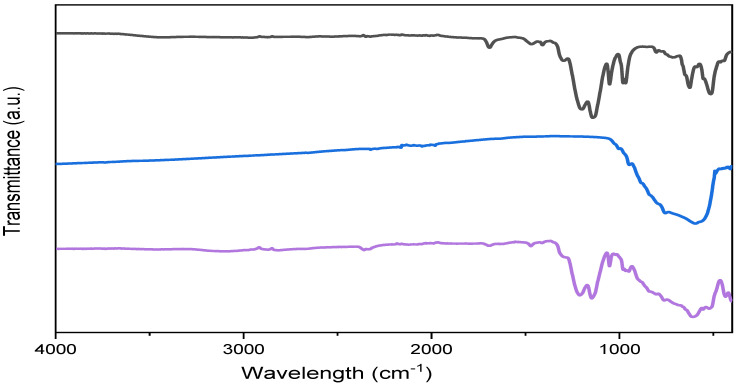
FTIR spectra of recast Nafion (black), tungsten oxide nanofiller calcinated at 700 °C (blue), and 15 wt% WO_3_–Nafion hybrid membrane (purple).

Figure 7 represents the FTIR spectra of the recast Nafion membrane, synthesised tungsten oxide, and 15 wt% tungsten oxide–Nafion hybrid membrane. The spectra of rNF showed the typical characteristic bands of recast Nafion. The bands at 1466 and 1410 cm^−1^, corresponding to undissociated –SO3H groups; ~1200 and 1142 cm^−1^ are attributed to C-F asymmetric and symmetric stretching. Whereas the band at approximately ~ 1050 cm^−1^ is assigned to the C–F stretching in the –CF2–CF(R)–CF3 group; ~974 cm^−1^, C–O–C stretching; additionally, the bands at 625, 517 cm^−1^ are believed to be attributed to the stretching of the C-S group and symmetric O-S–O bending [2,51,66]). On the other hand, the spectrum of monoclinic WO_3_ showed a broad band in the range of 400–1000 cm^−1^ attributed to the vibration modes of the W–O bond, which confirms the formation of tungsten oxide (600 cm^−1^, O–W–O stretching; 755 cm^−1^, W–O–W bending; 945 cm^−1^, W=O stretching; 997 cm^−1^, W–O stretching [67,68,69]). After the incorporation of the WO_3_, the bands at 1466 and 1410 cm^−1^, related to the undissociated –SO_3_H groups, diminished. Additionally, the bands at ~1200 and 1142 cm^−1^ slightly shifted to a higher wavenumber, which could be attributed to the change in the chemical surroundings. Moreover, the bands representing the stretching of the C–S group and the symmetric bending of the O–S–O merged in the broad band of the monoclinic WO_3_.

### 3.2. Contact Angle, Water Uptake, Swelling Ratio, Ion Exchange Capacity, and Hydration Degree

**Figure 8:**
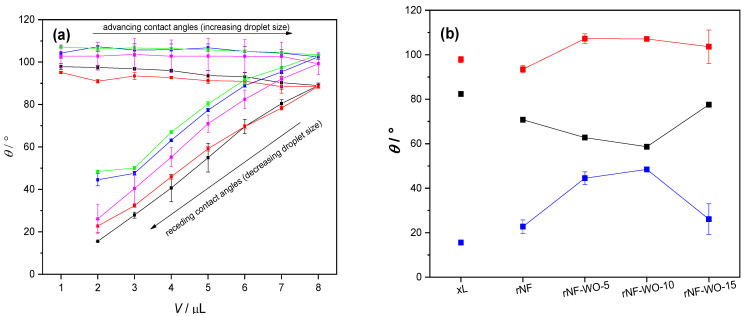
(**a**) Mean advancing and receding CAs with standard errors of three parallel measurements as a function of droplet volume, Nafion XL commercial membrane (black), recast Nafion membrane (red), Nafion membrane with 5, 10, and 15 wt% tungsten oxide (blue, green, and purple, respectively); (**b**) Maximum mean advancing (*θ*_max_—red) and minimum mean receding (*θ*_min_—blue) CAs with standard errors of three parallel measurements, and the CA hysteresis (Δ*θ* = *θ*_max_ − *θ*_min_—black).

Generally, the performance of the polymer electrolyte membranes relies on the hydration and hydrophilicity of the membrane. Therefore, the surface hydrophilicity was determined by water contact angle measurements. As argued before, differences in the wetting properties of surfaces are best revealed by measuring both the advancing and receding contact angles [57,70,71]. Representative images of water droplets in advancing, respectively receding stages with corresponding CAs, one per each sample type, are presented in the Supplementary Materials, Figure S1. Figure 8a summarises all CA data as the same volume means with standard errors. For all samples, advancing CAs are practically independent of the droplet volume, while receding angles decrease steeply with decreasing volume. The wetting properties of the recast Nafion and hybrid membranes were found to be slightly different from that of the commercial Nafion XL. Figure 8b shows the maximum mean advancing (*θ*_max_) and minimum mean receding (*θ*_min_) CAs with standard errors of three parallel measurements, and the CA hysteresis (Δ*θ* = *θ*_max_ − *θ*_min_). Except for the non-WO_3_-loaded sample (rNF), all hybrid–WO_3_ membranes exhibited 6–12° higher maximum CAs than the commercially available Nafion XL membrane. Similarly, all recast membranes, even the non-WO_3_-loaded one, exhibited 7–34° higher minimum CAs. Consequently, the CA hysteresis of the recast hybrid membranes was 10–24° lower than that of the Nafion XL.

**Figure 9:**
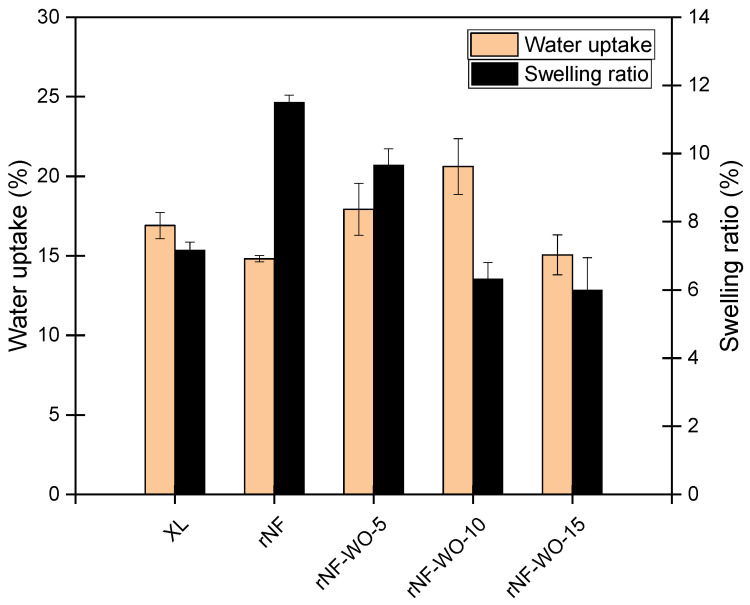
Hydration properties of the commercial, recast, and hybrid Nafion membranes.

**Figure 10:**
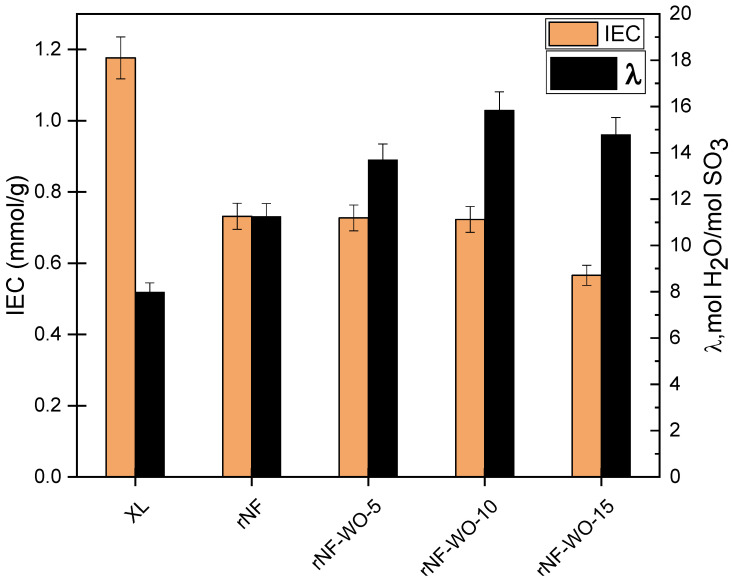
Ion exchange capacity and hydration degree analysis of the commercial, rNF, and rNF–WO_3_ hybrid membranes.

Thus, apparently, the addition of WO_3_ resulted in a hydrophobicity *increase* of the membranes. The hydrophobicity clearly increased with the added WO_3_ content up to 10%, where it broke down, but even the hydrophobicity of the 15% sample was found to be higher than that of the unloaded sample rNF. These results may appear somewhat surprising and counterintuitive, since the intrinsically hydrophilic WO_3_ filler (with literature reported water CAs, depending on the preparation parameters, ranging from 4 to 104°, typical values being around 30°; Supplementary Materials, Table S1) was expected to *decrease* the overall hydrophobicity of the membranes, and thus contribute to an improved wetting. This contradiction can be resolved, however, by assuming that the WO_3_ filler is incorporated in the bulk of the membrane, but not at its surface, while it still changes the orientation and/or density of the polymer chains at the surface. This explanation is supported by the fact that, although the surface hydrophobicity was found to increase with WO_3_ content, implying a decreasing surface wettability, application-relevant bulk membrane properties such as water uptake (Figure 9) and hydration degree (Figure 10) all increased with the increasing WO_3_ content (up to 10%, where showed the same sudden drop as CAs). CAs are related to the wettability of the surface but tell nothing about the processes undergoing in the bulk.

Again, the results point out the importance of measuring not only the advancing, but also the receding CAs: by ignoring the receding CAs, all of which are much smaller than 90°, and considering only the advancing CAs, all of which are all larger than 90°, all samples could be misjudged as hydrophobic, and their water uptake and retention properties could not be explained. Instead, advancing and receding CAs together suggest that the membrane surfaces can be regarded as multicomponent systems in terms of wetting, consisting of at least one hydrophobic and at least one hydrophilic component, which manifest in the advancing, respectively receding water CAs.

Water uptake and swelling ratio are usually used to describe the hydration behaviour of membranes. From an application point of view, both good water uptake and a low swelling ratio are desirable. The influence of different tungsten oxide nanofiller contents on the water uptake and swelling ratios of the membranes in DI water at room temperature is presented in Figure 9. The recast membrane exhibited good water uptake; however, it also showed a high swelling ratio, which may lead to a higher hydrogen crossover rate. The water uptake increased with the increasing the WO_3_ nanofiller content up to 10 wt. %, then it slightly decreased. Contrarily to the increase of water uptake, the swelling ratio decreased with the increasing WO_3_ content, and dropped significantly below the level of XL already at 10% nanofiller content, while the water uptake was still high. As compared to the unfilled Nafion membranes, the hybrid membrane with 10 wt% tungsten oxide is a more ideal material in terms of both water uptake and swelling ratio. Nevertheless, at too high a WO_3_ content (15 wt%), the water uptake drops as well. This phenomenon can be attributed to a masking effect of the SO_3_H groups upon the addition of a higher nanofiller amount, inhibiting the water uptake of the polymer chains. In addition, there is also a possibility for the aggregation and formation of a thick nanofiller layer on the outer surface of the membrane [3,41]. Such an explanation is consistent with the fact that rNF–WO-15 showed slightly lower advancing and receding contact angles than both rNF–WO-5 and rNF–WO-10. Nevertheless, it has to be emphasised again that, compared to the commercial Nafion XL membrane, rNF–WO-10 shows better performance, as it presents a higher water uptake while controlling the swelling ratio of the membranes, which restricts the hydrogen crossover of the membrane as well [55].

Generally, ion exchange capacity describes the number of the exchangeable groups in the membranes, and therefore plays an essential role in the membrane proton conductivity. The IEC and degree of hydration of the membranes are shown in Figure 10. The IEC of the recast Nafion membranes and the Nafion–WO_3_ membranes is approximately the same up to 10 wt% nanofiller content. However, the incorporation of higher WO_3_ content to the membranes led to a decrease in the IEC. This might be due to the presence of the smaller nano-WO_3_ particles inside the Nafion conducting channels, which prevent the SO_3_H groups from acting as conductive groups. Another explanation is that, upon the addition of a higher nanofiller content, the ratio of sulfonic acid groups in the hybrid membrane decreases because of the diluting effect of the nanofiller, therefore the number of the SO_3_H groups decreases, and consequently so does the IEC. On the other hand, it is recognised that the degree of hydration increased up to 10 wt% nanofiller content, and then it decreased, probably due to the excess nanofiller inhibiting the water uptake in the polymer chain. A similar trend has already been reported [41,64].

### 3.3. Membrane Conductivity and In Situ Single Cell Testing

The proton conductivity values of the nano-hybrid membranes and the recast Nafion membrane at 25 °C were calculated from the impedance spectra and the Nyquist plot, where the total resistance of the MEA can be calculated from Equation (8):

(8) $$R_{MEA}=R_{membrane}+2\times R_{electrode}\;$$

The same amount of catalyst at both the anode and cathode sides was used, therefore the resistance of both is equally $R_{\mathrm{electrode}}$= 0.0228 Ω. Table 1 presents the calculated conductivity values for the recast Nafion and nano-hybrid membranes. In accordance with the literature, the proton conductivity of the hybrid membranes up to 10 wt% is higher than that of the recast Nafion membrane. The proton conductivity initially increased up to 10 wt% tungsten oxide content and then decreased with the increasing nanofiller content. This trend is in good agreement with the contact angle, water uptake, IEC, and hydration degree changes with increasing WO_3_ content. Obviously, there is a trade-off in the nanofiller content. Initially, at a low WO_3_ content, it serves as a water and probably a proton reservoir as well. At a higher WO_3_ content, however, it masks the sulfonic acid groups and interrupts the proton conduction channels in the Nafion matrix or leads to the formation of proton conduction pathways with high tortuosity [22,45].

In order to evaluate the performance of the different used membranes, including the recast Nafion membranes, the hybrid–WO_3_-containing membranes with the different concentrations, and comparing their performance as PEMs with the commercially available Nafion XL membrane, the voltage–current density polarization curves were measured. The power density and current density over different membranes were calculated at 0.4 V, as roughly at this voltage maximum in power density was obtained. The other approach generally accepted in the related art applied for comparison of performances is to fix the voltage at 0.65 V, which corresponds to 50% electrical efficiency [72]. In terms of the relative order of performance between membranes, the two approaches generally do not lead to a difference [73,74].

**Figure 11:**
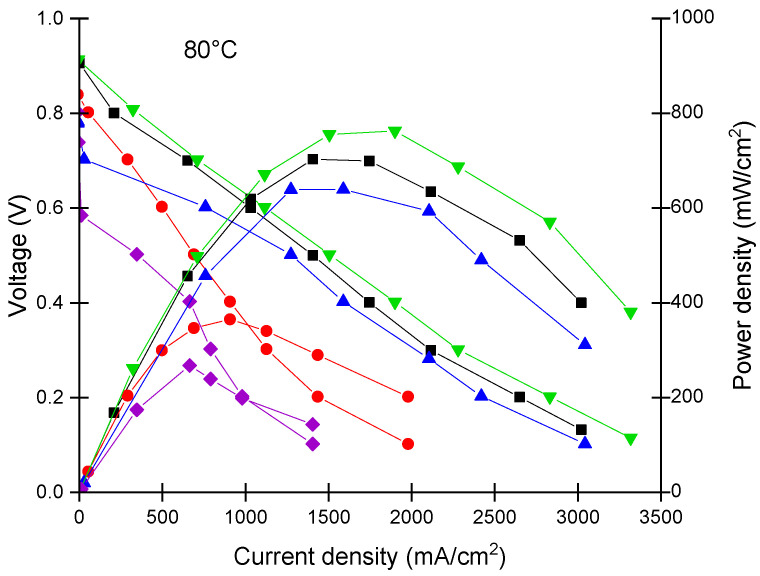
Polarisation and power density curves of the membranes at 80 °C and relative humidity of 50% and 30% for H_2_ and O_2,_ respectively. Nafion XL commercial membrane (black), recast Nafion membrane (red), and Nafion membrane with 5, 10, and 15 wt% tungsten oxide (blue, green, and purple, respectively).

Figure 11 shows the performance of the single fuel cell for the recast and hybrid membranes compared to that of the commercial Nafion XL membrane at the conventional operating temperature (80 °C) and relative humidity of 50% and 30% for the H_2_ and O_2_ stream, respectively. The membrane with 10 wt% WO_3_ presented superior performance even when compared to the commercial membrane. Additionally, both the rNF–WO-5 and rNF–WO-10 hybrid membranes exhibited higher performance than the rNF membrane, which can be attributed to the water-retaining ability of WO_3_. Moreover, unsurprisingly, the 15 wt% membrane showed the lowest performance; this can be explained by the above-discussed possibility of the sulfonic acid groups being blocked by the tungsten oxide nanofiller, causing a decrease in the ion conductivity and, therefore, the lower membrane performance.

**Figure 12:**
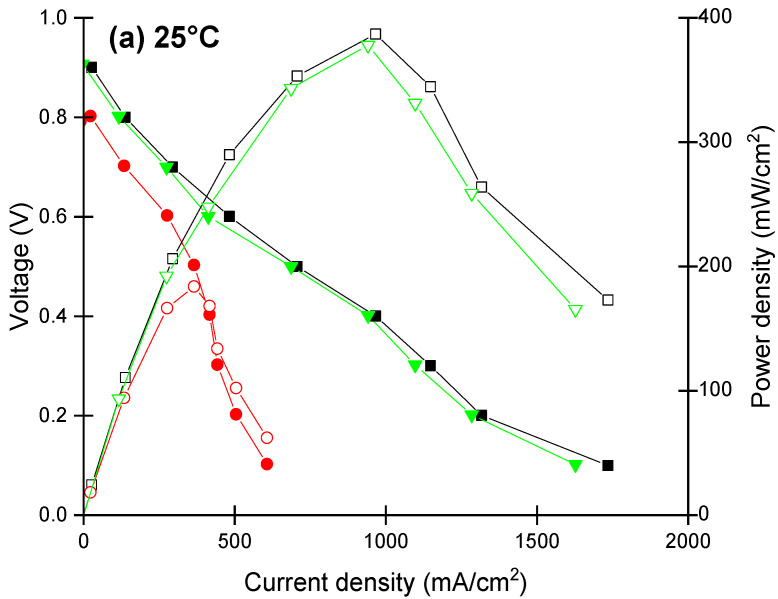

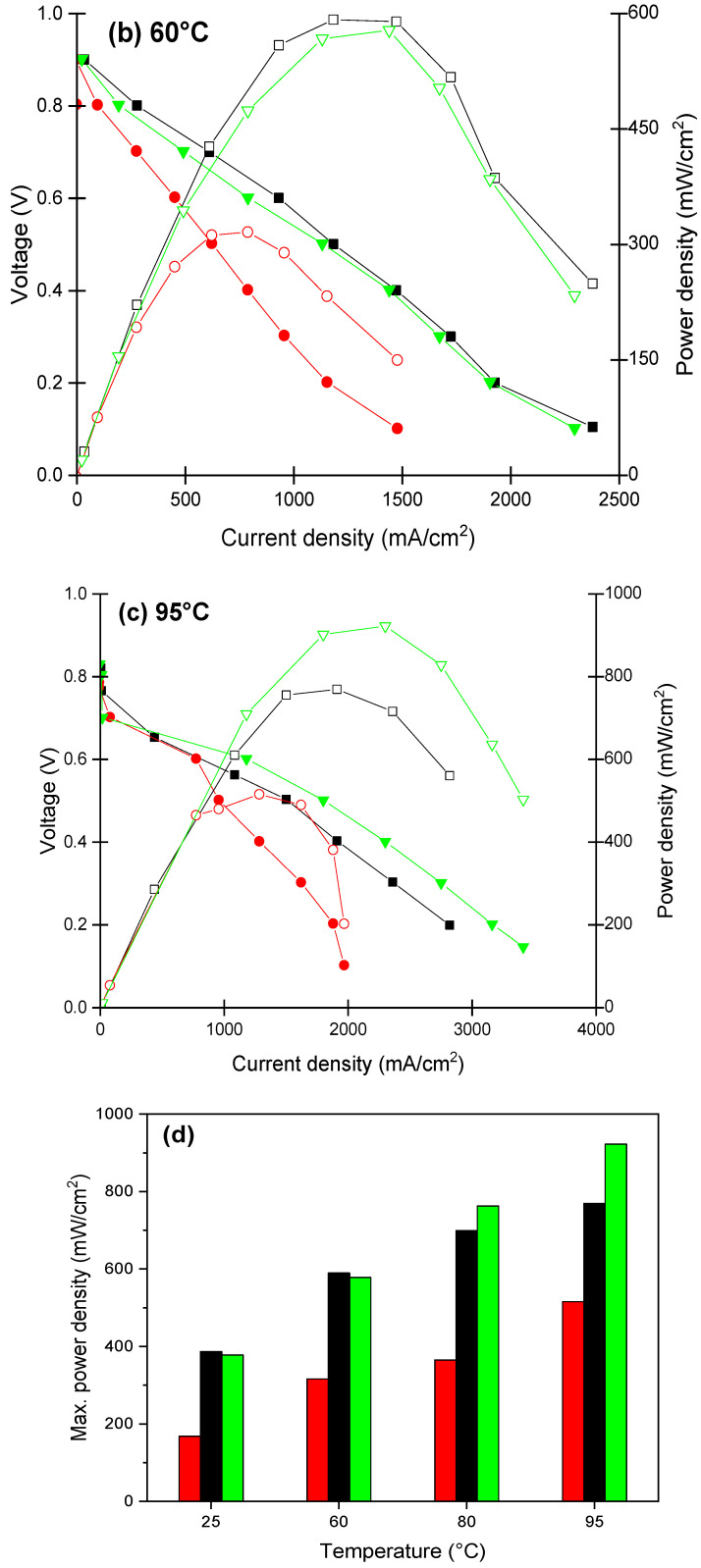

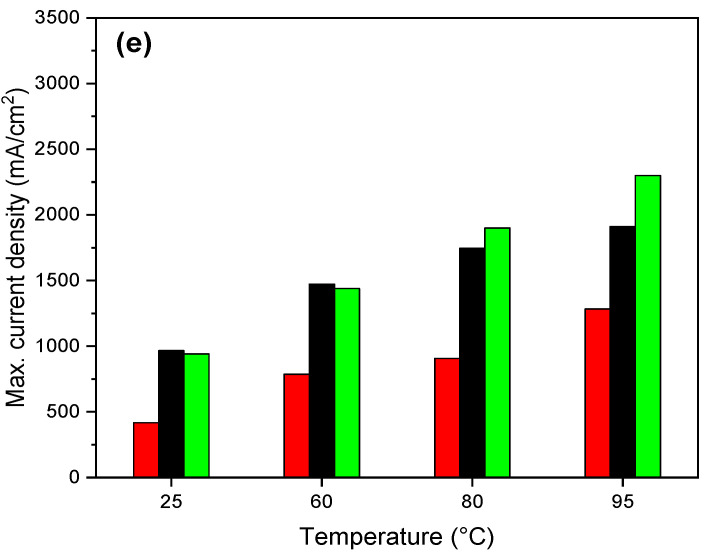
Influence of temperature on the single cell performance. Polarisation and power density curves at (**a**) 25, (**b**) 60, and (**c**) 95 °C, respectively, (**d**) maximum power density and (**e**) maximum current density at 0.4 V. Recast Nafion membrane (red), Nafion XL commercial membrane (black), and Nafion membrane with 10 wt% tungsten oxide (green).

Figure 12 shows the single cell performance of the rNf–WO-10 hybrid membrane compared to that of the commercial and recast Nafion membranes at different temperatures. At 25 and 60 °C, the Nafion XL membrane showed better performance than the hybrid one. At these low temperatures, the water content of the membranes is probably high enough to boost the conductivity, thus the performance is not controlled by the humidification. At higher temperatures (80 and 95 °C), however, the rNF–WO-10 membrane showed better performance than both rNF and Nafion XL, as the unfilled membranes can lose water more easily, leading to increased membrane resistance and shrinkage of the membrane. The rNF and Nafion XL have higher resistance at a higher temperature compared to the rNF–WO-10 hybrid membrane. This highlights the effect of WO_3_ on improving the cell performance and its ability to retain more water, therefore ensuring that the proton conductivity will not be deteriorated.

Figure 12d,e show the maximum power densities and maximum current densities obtained at 0.4 V based on the New European Driving Cycle protocol [71]. All three membranes showed improving performance with increasing temperature. At lower temperatures, the Nafion XL membrane showed slightly higher performance than the rNF–WO-10. However, at the higher temperatures, the rNF–WO-10 showed the highest maximum power density, reaching 0.76 and 0.922 W/cm^2^ at 80 and 95 °C, respectively. The maximum current of rNF–WO-10 is the highest among the three membranes when operating at 80 °C and 95 °C, with values of 1.89 and 2.29 A/cm^2^, respectively.

## 4. Conclusions

Nanosized monoclinic tungsten oxide (WO_3_) was produced using a hydrothermal process at an annealing temperature of 700 °C. WO_3_ was used as a nanofiller in Nafion-based membranes to allow higher hydration levels in the membrane during the fuel cell process at elevated temperatures and to maintain the ionic conductivity at low humidity conditions.

Recast Nafion and Nafion–tungsten oxide hybrid membranes with three different nanofiller concentrations were fabricated using the solution casting method. The XRD analysis showed that incorporation of a small amount of WO_3_ (up to 10 wt%) enhanced the crystallinity of the membranes, while increasing the nanofiller content led to a decrease of the main characteristic peak of Nafion at 2θ=17.6°, indicating a possible corruption of the polyfluorocarbon chain. TGA revealed that the incorporation of the nanofiller increased the decomposition temperature and the weight residue of the membranes.

Compared to both the recast Nafion and the commercially available Nafion XL membrane, the hybrid membranes exhibit higher mechanical stability, improved water uptake and swelling properties, as well as higher hydration degree up to 10 wt% filler content. The slightly higher contact angles indicating slightly decreased hydrophilicity did not deteriorate these properties. The surface wettability might affect the rate of water uptake only, but not the amount of water stored in the bulk phase of the membranes. Additionally, all the hybrid membranes showed a lower swelling ratio than the recast membrane, with a minimum value at 15 wt% WO_3_ composition. The ion exchange capacity of the new hybrid membranes was smaller than that of Nafion XL, being practically equivalent to that of the recast Nafion.

In situ single cell performance of the hybrid membrane with 10 wt% WO_3_ was the highest compared to both rNF and XL membranes at the conventional temperature (80 °C) and relatively low humidity of 50% H_2_ and 30% O_2_ stream. The performance of the rNF–WO-10 membrane was further improved at a higher temperature (95 °C). The maximum power and current density of 0.4 V were achieved by rNF–WO-10, 0.76 W/cm^2^ and 1.89 A/cm^2^ when operating at 80 °C, and 0.922 W/cm^2^ and 2.29 A/cm^2^ at 95 °C, respectively. A strong correlation between the water-retaining properties of WO_3_ and single cell performance has been observed. There is an optimum concentration of tungsten oxide in the hybrid membrane: at too high a nanofiller content, the favourable water-retaining properties of WO_3_ are compromised due to its negative effect on the sulfonic acid groups.

In conclusion, the hybrid membrane with 10 wt% WO_3_ offers an enhanced performance while allowing operating temperatures as high as 95 °C and maintaining the mechanical and dimensional stability at a low humidity.

## Figures and Tables

**Table 1:** Membrane thickness (t), resistance (R), and proton conductivity (*σ*) values of recast Nafion and hybrid membranes at 25 °C and 100% RH.

	*t* (µm)	*R*_MEA_ (Ω)	*R*_membrane_ (Ω)	*σ* (mS cm^−1^)
rNF	~27.8	0.081	0.035	4.89
rNF–WO-5	43	0.080	0.035	7.72
rNF–WO-10		0.058	0.012	22.16
rNF–WO-15		0.088	0.042	6.34

## Data Availability

The data presented in this study are available on request from the corresponding author.

## Supplementary Materials

The following supporting information can be downloaded at: https://www.mdpi.com/article/10.3390/polym14122492/s1, Figure S1: Representative advancing (*θ*a) and receding (*θ*r) water contact angles of the membranes; Table S1: Reported (static) water contact angles of different WO_3_ layers. References [75,76,77,78,79,80,81,82,83,84,85,86,87] are cited in the supplementary materials.
