# Supplementary material for: Development of WO3–Nafion Based Membranes for Enabling Higher Water Retention at Low Humidity and Enhancing PEMFC Performance at Intermediate Temperature Operation
Source: Polymers (Basel). 2022 Jun 19;14(12):2492. doi: 10.3390/polym14122492 (PMC9227791; doi:10.3390/polym14122492)
Supplement: Supplementary file 1 [file polymers-14-02492-s001.zip › polymers-1757756-supplementary.pdf]

Supporting Information

Development of WO_3-Nafion based membranes for enabling higher water retention at low humidity and enhancing PEMFC performance at intermediate temperature operation.

Asmaa Selim[a,b], Gabor Szijjarto[a], Loránd Románszki[c], András Tompos[a]

aRenewable Energy Group, Institute of Materials and Environmental Chemistry, Research Centre for Natural Sciences, H-1117 Budapest Magyar tudósok körútja2, Hungary
bChemical Engineering and pilot plat Department, Engineering and Renewable Energy Research Institute National Research Centre, 33 El Bohouth Street, 12622 Giza, Egypt
cFunctional Interfaces Research Group, Institute of Materials and Environmental Chemistry, Research Centre for Natural Sciences, H-1117 Budapest Magyar tudósok körútja2, Hungary

Contact angle results

sample type	θ_a / °	θ_r / °
XL	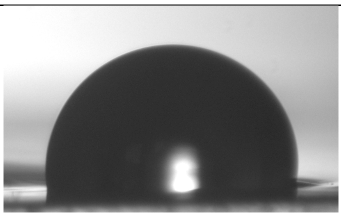<br>98.0	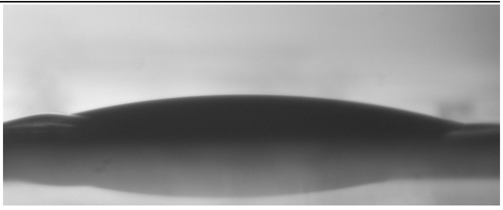<br>15.5
rNF	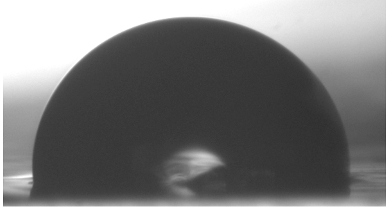<br>93.4	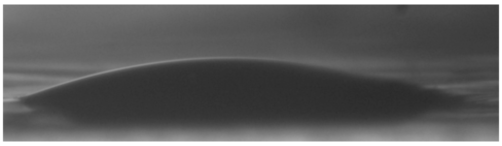<br>19.7
rNF + 5% WO_3	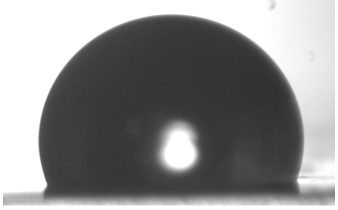<br>106.7	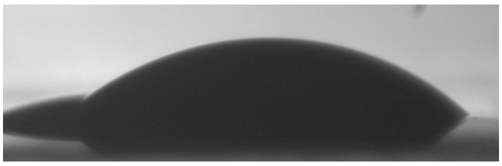<br>44.7
rNF + 7.5% WO_3	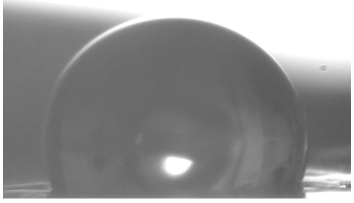<br>109.3	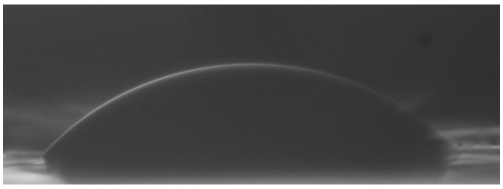<br>46.8
rNF + 10% WO_3	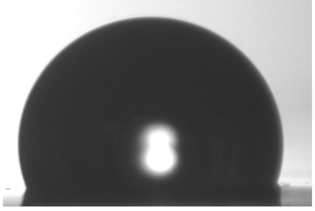<br>107.2	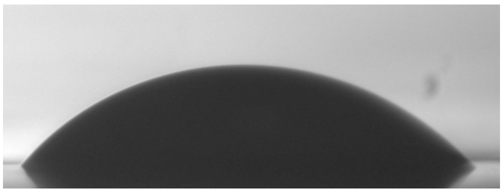<br>48.2
rNF + 15% WO_3	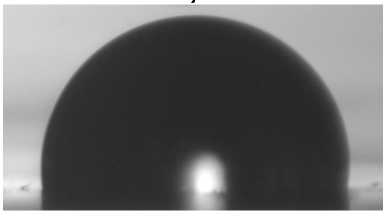<br>97.6	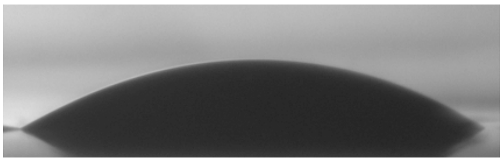<br>33.0

Figure S1. Representative advancing (θ_a) and receding (θ_r) water contact angles of the membranes.

WO_3 is known to be hydrophilic, with reported (static) water contact angles ranging from 4 to 104°, typically around ~30° (Table S1).

Table S1. Reported (static) water contact angles of different WO3 layers

type	water CA / °	remark	ref
sol–gel dip-coated	13–55	in function of the annealing temp.	Azimirad 2007; Naseri 2007
sol–gel dip-coated	97–104	5-15 multilayer	Raudoniene, 2018
thermally evaporated film	4–57	in function of the annealing temp.	Azimirad 2007
sol–gel spin-coated	26–30	further decreasing by long UV irradiation	Miyauchi M 2009
spray pyrolisis	28	nanoporous layer	Vlauduta C, 2008; Enesca, 2008
spray pyrolisis	58	dense layer	Vlauduta C, 2008; Enesca, 2008
pulsed spray pyrolysis	30		Enesca, 2008
sol–gel spin-coated	21–92	in function of the annealing temp.	Hemati, 2013
sol–gel	44	after being annealed at 450 °C for 1 h	Liu, 2018
spray pyrolisis	31		Vardhan, 2022
pulsed laser deposition	27–56	depending on the process temperature	Behbahani, 2013
spray pyrolisis	6–7		Vardhan, 2020
sol–gel spin-coated	16–36	on the top of VO_2 substrate layers; depending on process parameters	Top, 2018
reactive magnetron sputtering	5–73	depending on process temperature	Ramana, 2020

References

1. Azimirad R, Naseri N, Akhavan O, Moshfegh AZ, Hydrophilicity variation of WO3 thin films with annealing temperature, J. Phys. D: Appl. Phys. 40 (2007) 1134–1137.
2. Behbahani MA, Ranjbar M, Kameli P, Salamati H, Hydrogen sensing by wet-gasochromic coloring of $PdCl_2$(*aq*)/WO_3 andthe role of hydrophilicity of tungsten oxide films, Sensors and Actuators B 188 (2013) 127– 136.
3. Enesca A, Duta A, Tailoring WO3 thin layers using spray pyrolysis technique, Phys. Stat. Sol. (c) 5 (2008) 3499–3502.
4. Hemati A, Allaf B M, Ranjbar M, Kameli P, Salamati H, Gasochromic tungsten oxide films with $PdCl_2$ solution as an aqueous Hydrogen catalyst, Solar Energy Materials & Solar Cells 108 (2013) 105–112.
5. Liu Y, Yang Y, Liu Q, Li Y, Lin J, Li W, Li J, The role of water in reducing WO_3 film by hydrogen: Controlling the concentration of oxygen vacancies and improving the photoelectrochemical performance, Journal of Colloid and Interface Science 512 (2018) 86–95.
6. Miyauchi M, Shibuya M, Zhao ZG, Liu Z, Surface wetting behavior of a WO_3 electrode under light-irradiated or potential-controlled conditions, J. Phys. Chem. C 2009, 113, 10642–10646.
7. Naseri N, Azimirad R, Akhavan O, Moshfegh AZ, The effect of nanocrystalline tungsten oxide concentration on surface properties of dip-coated hydrophilic WO_3–SiO_2 thin films, J. Phys. D: Appl. Phys. 40 (2007) 2089–2095.
8. Ramana CV, Battu AK, Dubey P, Lopez GA, Phase-control-enabled enhancement in hydrophilicity and mechanical toughness in nanocrystalline tungsten oxide films for energy-related applications, ACS Appl. Nano Mater. 2020, 3, 3264–3274.
9. Raudoniene J, Laurikenas A, Kaba MM, Sahin G, Morkan AU, Brazinskiene D, Asadauskas S, Seidu R, Kareiva A, Garskaite E, Textured WO3 and WO3:Mo films deposited from chemical solution on stainless steel, Thin Solid Films 653 (2018) 179–187.

10. Top I, Binions R, Sol C, Papakonstantinou I, Holdynski M, Gaiaschi S, Abrahams I, Improved thermochromic properties in bilayer films of VO_2 with ZnO, SnO_2 and WO_3 coatings for energy efficient glazing, J. Mater. Chem. C, 2018, 6, 12555.
11. Vardhan RV, Kumar S, Mandal S, A facile, low temperature spray pyrolysed tungsten oxide (WO_3): an approach to antifouling coating by amalgamating scratch resistant and water repellent properties, Bull. Mater. Sci. (2020) 43:281.
12. Vardhan RV, Kumar S, Mandal S, Fabrication of minimal capital-intensive scratch-resistant and hydrophobic tungsten oxide film on stainless steel through spray pyrolysis, Surf Interface Anal. 2022 1–14.
13. Vladuta C, Andronic L, Visa M, Duta A, Ceramic interface properties evaluation based on contact angle measurement, Surface & Coatings Technology 202 (2008) 2448–2452.
